# Pelvic Irradiation: A Rare Cause of Concomitant Radiation Cystitis and Uretero-Iliac Artery Fistula Causing Gross Hematuria and Hemorrhagic Shock

**DOI:** 10.7759/cureus.25774

**Published:** 2022-06-09

**Authors:** Maleeha Saleem, Karan H Pahuja, Alex Arnouk

**Affiliations:** 1 Internal Medicine, Saint Francis Medical Center, Trenton, USA; 2 Urology, Saint Francis Medical Center, Trenton, USA

**Keywords:** uretero-iliac artery fistula, provocative angiography, retrograde pyelogram, hemorrhagic cystitis, radiation cystitis

## Abstract

Uretero-iliac artery fistulas (UIAFs) are a rare cause of gross hematuria. They form as a result of poorly vascularized uretero-iliac adhesions and the resultant fibrosis and chronic inflammatory changes. Causes include previous pelvic surgery, radiotherapy, and chronic ureteral stenting. The presentation is usually intermittent massive gross hematuria with acute anemia and hemorrhagic shock. A high index of suspicion is warranted in patients with predisposing factors for prompt diagnosis and management as it may be associated with life-threatening hemorrhage. Due to the intermittent nature of symptoms, identification is not always apparent. Open surgical repair was the treatment of choice in the past. With advancements in interventional radiology techniques, endovascular stenting of the iliac artery and concomitant ureteral stenting is the current treatment of choice. We report a case of massive gross hematuria leading to hemorrhagic shock with underlying UIAF and predisposing risk factor of pelvic irradiation. Our case report describes the diagnostic challenges with associated comorbid conditions causing delays in successful management.

## Introduction

Uretero-iliac artery fistula (UIAF) is an uncommon but recognized clinical entity that occurs in patients with prior pelvic surgeries for malignancies, radiation exposures, indwelling ureteral stents, degenerative iliac artery disease, prior arterial reconstructive surgery, or arterial prosthetic grafts [1,2]. The communication typically involves the iliac artery and the ureter and is caused by chronic inflammatory changes, extensive fibrosis, and eventual necrosis of the ureteric and iliac artery walls leading to the development of a fistula [3]. The location is typically where the ureter crosses over the iliac artery as they are in the closest proximity at this point. The presentation is intermittent massive gross hematuria with associated acute anemia and/or hemorrhagic shock [4]. Angiography, particularly provocative angiography, remains the first-line diagnostic tool [5]. However, diagnosis can be elusive even when multiple imaging modalities are used because sensitivity is suboptimal [2]. The treatment of UIAF has evolved and now the vascular defect can be repaired primarily using embolization, ligation, or endovascular stenting [6]. A high mortality rate of approximately 7-23% has been described in the literature [4,7]. We report a case of UIAF in a patient with a history of pelvic irradiation. Here, we discuss the diagnostic challenges and how associated hemodynamic instability and comorbid conditions can lead to delayed management.

## Case presentation

An 82-year-old male with a medical history significant for hypertension, hyperlipidemia, type 2 diabetes, prostate cancer, chronic obstructive pulmonary disease, and coronary artery disease status post placement of five cardiac stents, most recent in December 2022, presented to the emergency department with abdominal pain, gross hematuria, and altered mentation. About a week ago, he had experienced burning urination associated with hematuria, and his primary care doctor empirically treated him with antibiotics for a presumed urinary infection. Over the next few days, he developed worsening gross hematuria and severe lower abdominal pain that was mainly in the hypogastric area, aching in nature, 10/10 in severity, and non-radiating with no aggravating or relieving factors. The patient was found to have acute urinary retention that was relieved with Foley catheter placement of 1,000 mL of bloody urine containing blood clots. The patient had a history of prostate cancer and received radiation therapy about three years prior. On physical examination, he was in mild distress. The abdomen was distended and tender to palpation in the suprapubic region. Initial vitals showed a blood pressure of 121/63 mmHg with a heart rate (HR) of 92 beats per minute, respiratory rate (RR) of 16 breaths per minute, and oxygen saturation of 98% on room air. Initial labs demonstrated a slightly elevated creatinine, normal white cell count, macrocytic anemia with hemoglobin of 9.7 g/dL from a baseline of 12 g/dL (two months prior) with a normal international normalized ratio (INR), and a normal platelet count (Table 1). Urinalysis showed positive nitrites, moderate leukocyte esterase, few white blood cells (WBCs), and greater than 100 red blood cells (RBCs), as shown in Table 2.

**Table 1:** Laboratory test results. BUN: blood urea nitrogen; WBC: white cell count; Hb: hemoglobin; Hct: hematocrit; MCV: mean corpuscular volume; INR: international normalized ratio

Lab parameters (units)	Result	Reference range
BUN (mg/dL)	16	7–25
Creatinine (mg/dL)	1.35	0.70–1.30
WBC (k/mL)	8.8	3.8–10.2
Hb (g/dL)	9.7	12.9–16.7
Hct (%)	29.4	39–48
MCV (fL)	98.9	79–96
Platelet count (k/mL)	248	130–400
INR	1.3	1–1.5

**Table 2:** Urinalysis findings. HPF: high-power field; RBC: red blood cell; WBC: white blood cell

Urinalysis	Result	Normal value
Color	Red	
Clarity	Turbid	
Glucose (mg/dL)	Negative	Negative
Bilirubin (mg/dL)	Negative	Negative
Ketones (mg/dL)	Negative	Negative
Specific gravity	1.030	1.005–1.030
Blood	Moderate A	Negative
pH	7.5	4.0–8.0
Total protein (mg/dL)	100 A	Negative
Urobilinogen (EU/dL)	0.2	0.2–1.0
Nitrites	Positive A	Negative
Leukocyte esterase	Moderate A	Negative
WBC/HPF	5–10 A	0–2
RBC/HPF	>100 A	0–2
Bacteria/HPF	Moderate A	Negative

**Figure 1:**
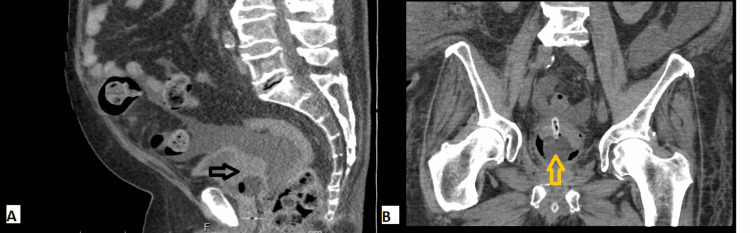
Sagittal view (A) and coronal view (B) of CT abdomen/pelvis showing density surrounding the Foley catheter balloon within the urinary bladder measuring up to 5.1 cm, as indicated by the black arrow, possibly representing blood clot and a hyperdensity within the dependent portion of the urinary bladder which may represent hemorrhage, as indicated by the yellow arrow. Increased gas is seen within the urinary bladder. Wall thickening within the urinary bladder with adjacent stranding is noted along with a moderate amount of ascites. CT: computed tomography

Computed tomography (CT) of the abdomen and pelvis with intravenous (IV) contrast showed prostatomegaly with fiducial markers in place. There was thickening of the urinary bladder wall along with densities in the bladder consistent with clots (Figures 1A, 1B). Cirrhosis was also noted with significant ascites. There was no evidence of renal masses, hydronephrosis, or nephrolithiasis.

The patient’s dual antiplatelets (aspirin and clopidogrel) were held due to active hematuria, and he was managed with antibiotics and intravenous fluids. He was evaluated by Urology who suggested that the hematuria was likely related to radiation cystitis or hemorrhagic cystitis from a urinary tract infection. He underwent bedside catheter exchange with a 22-French three-way catheter and manual irrigation of multiple clots. He was subsequently started on continuous bladder irrigation (CBI) with near resolution of the hematuria. Overnight, the patient again developed hematuria although previously he had started producing clear urine on CBI, resulting in worsening blood loss anemia with a hemoglobin of 6.6 g/dL requiring two units of packed red blood cells (PRBCs) after which hemoglobin improved to 8.3 g/dL.

Early next morning, he was taken to the operating room for a cystoscopy. The cystoscopy revealed a loosely organized clot of about 100 mL within the urinary bladder that was evacuated. The patient’s bladder neck was normal but there was evidence of radiation-type changes along the left and right bladder neck as well as the trigone. He underwent fulguration of friable areas of the bladder mucosa. The patient was restarted on CBI, with clear urine noted, following the procedure.

**Figure 2:**
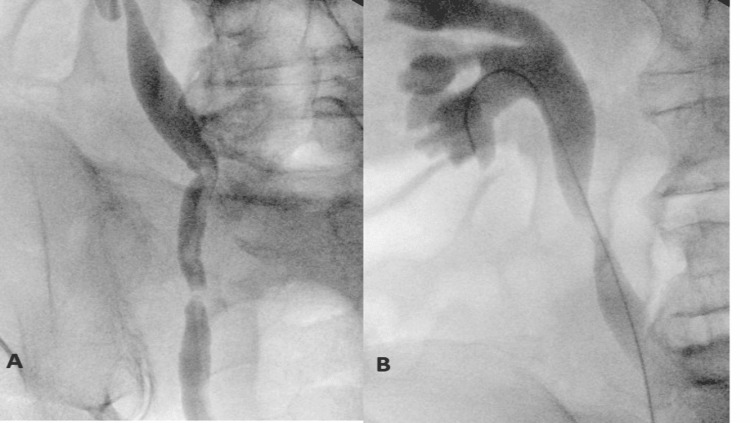
Retrograde pyelogram demonstrating (A) filling defect in the region of the mid-to-distal right ureter and multiple hyperdense filling defects within the right ureter suggestive of blood clot and (B) mild right-sided hydroureteronephrosis without a clear renal source visualized.

Several hours post-procedure, the patient underwent large-volume, ultrasound-guided, right-sided paracentesis with the removal of nearly 5,000 mL of ascitic fluid. After paracentesis, there was a return of gross hematuria, frank red with clots, which did not cease despite catheter change, traction on the bladder neck with a 40 cc balloon, and manual bladder irrigation. There was a high suspicion of an upper-tract fistula. That evening, the patient was taken back to the operating room for clot evacuation and control of bleeding. Intraoperatively, the patient was noted to have frank blood with clots draining from the right ureteral orifice, concerning for a ureteral-iliac fistula. There was no evidence of arterial bleeding within the bladder. Retrograde pyelography demonstrated a filling defect in the region of the mid-to-distal right ureter at the point where the ureter crosses the iliac artery, as well as multiple hyperdense filling defects consistent with a thin ureteral blood clot (Figure 2).

A 6-French, double-J, right-sided ureteral stent was placed. For a brief period, it did help in improving the bleeding. However, with continuous irrigation, a significant amount of hematuria recurred. After a discussion of cystoscopy findings with an interventional radiologist, the patient was scheduled for an emergent CT angiography of the abdomen and pelvis.

**Figure 3:**
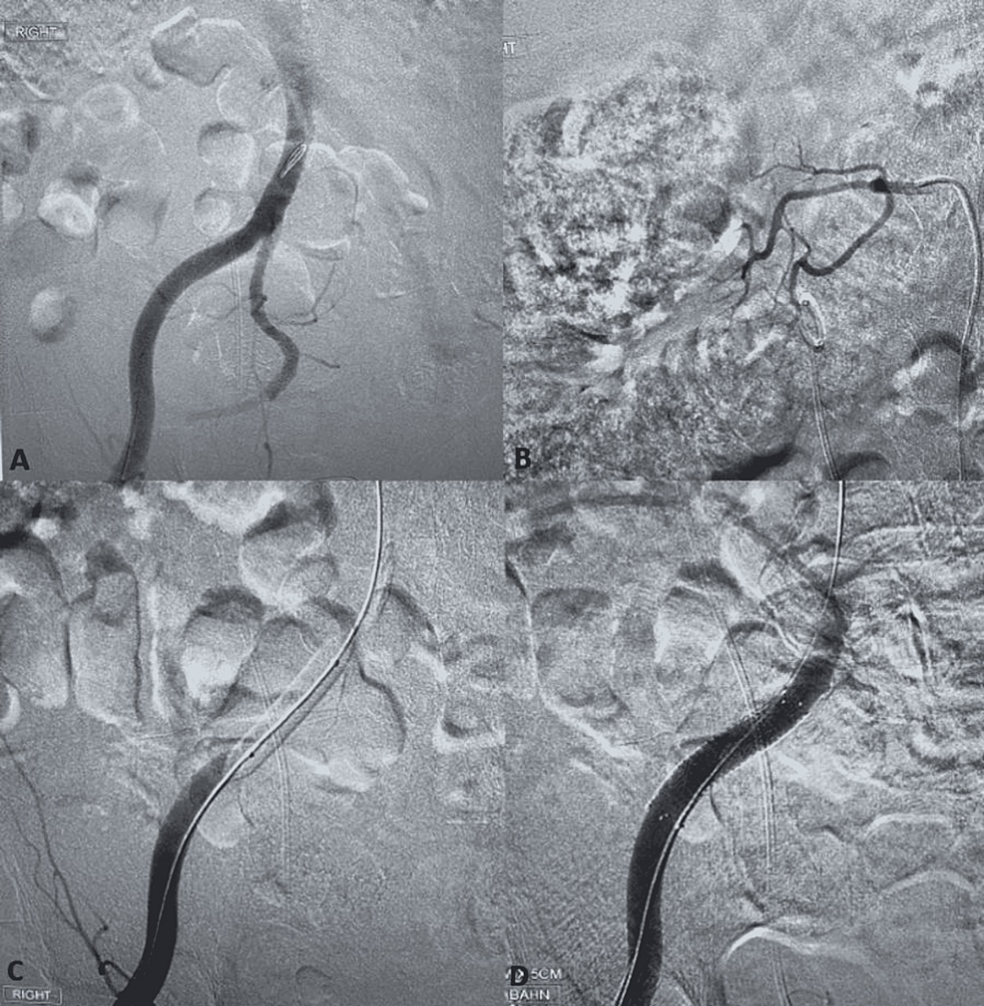
A and B: No evidence of extravasation in the right external/internal iliac or common iliac artery or right renal artery on arteriography. C and D: An 11 mm × 50 mm covered iliac stent was deployed in the external iliac artery across the region where the ureter crosses the iliac artery.

CT angiography did not clearly demonstrate active extravasation from the iliac artery, and no other obvious source was noted. His hemoglobin dropped to 7.3 g/dL from 8.3 g/dL, INR was elevated at 1.5, and he developed thrombocytopenia with a platelet count of 119 (k/mL) requiring four PRBCs, one unit of platelets, and one unit of fresh frozen plasma, and also required hemodynamic support due to hypotension with a mean arterial pressure (MAP) of 50 mmHg with triple pressor support with norepinephrine (40 µg/minute), phenylephrine (150 µg/minute), and vasopressin (0.04 U/minute) while in ICU. The patient developed hemorrhagic shock due to ongoing gross hematuria. The patient was kept intubated post-procedure. After transfusion of PRBCs, his hemoglobin did improve to 7.9 g/dL but again dropped to 7 g/dL. Due to interventional radiology resource limitations of iliac artery stenting, he was transferred to a nearby hospital for diagnostic angiography. He underwent right iliac artery stenting due to suspected UIAF. The patient underwent a right aorto-iliac arteriogram as well as selective catheterization of the right renal artery with arteriography. There was no evidence of arterial ureteral fistula or extravasation within the common, external, and internal iliac artery. Moreover, no extravasation was noted within the right renal artery on interrogation. An 11 mm × 50 mm covered Viabahn stent was deployed across the external iliac artery in the region of the ureter (identified by previously placed stent) empirically given retrograde pyelogram findings noted by urology (Figure 3).

**Figure 4:**
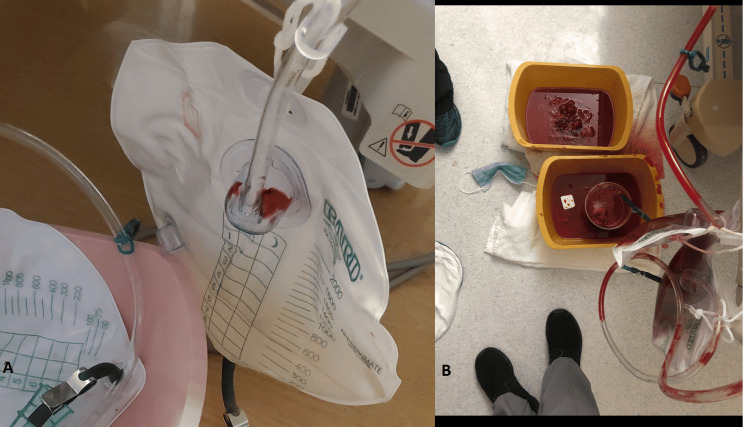
Foley catheter drainage bag tubing (A) clear urine on low rate CBI after iliac artery stenting compared with (B) massive gross hematuria pre-procedure. CBI: continuous bladder irrigation

There was an immediate resolution of the patient’s gross hematuria after the placement of the endovascular stent, as shown in Figure 4.

There were no immediate complications due to his urologic or interventional radiology procedures. His hematuria did not recur. He remained in hemorrhagic shock over the next few days requiring ventilatory support and hemodynamic support with pressors. He developed stress cardiomyopathy/non-ST-elevation myocardial infarction during this time. A two-dimensional echocardiogram showed diffuse global hypokinesis of the left ventricle with an estimated ejection fraction of 42%, elevated troponins that peaked at 9.084 ng/mL, and elevated NT-pro-brain natriuretic peptide (pro-BNP) of 36,920 pg/mL.

Although his hemodynamic parameters improved, given his stress cardiomyopathy, cirrhosis, advanced age, and large volume fluid shifts, he was not a candidate for coronary revascularization. He was unable to be weaned off of ventilatory support. His family ultimately decided to proceed with comfort care code status, and the patient expired eight days post-procedure, once terminally extubated.

## Discussion

UIAF is an uncommon cause of hematuria, with severity ranging from microscopic, intermittent, or life-threatening continuous hemorrhage [3,8]. It typically presents in patients with a history of chronic ureteral stenting, pelvic surgery, malignancy or radiation, and vascular diseases [8,9]. In cases of pelvic radiation therapy, the integrity of vasa vasorum can be disrupted, leading to chronic inflammation and increased susceptibility to arterial wall necrosis and rupture, eventually causing the formation of a fistula [9].

Our patient had two predisposing risk factors for the development of UAIF including pelvic irradiation for prostate cancer and recent paracentesis. He presented with sudden-onset gross hematuria which was initially thought to be secondary to infectious hemorrhagic cystitis and/or radiation cystitis. UIAF was not initially suspected due to findings of mild radiation-type changes in the bladder mucosa. A high index of suspicion began once hematuria became persistent and incessant after paracentesis causing hemorrhagic shock along with the demonstration of pulsatile efflux of bloody urine from the right ureteral orifice on cystoscopy. It was only after the paracentesis due to tense ascites, that the intra-abdominal pressure change possibly led to increased blood flow via the fistula, manifesting as severe continuous gross hematuria.

Diagnosis of a UIAF is difficult to establish and often requires more than one diagnostic tool [3]. Vascular surgery/interventional radiology should be involved early in the management [10]. Angiography, abdominal CT imaging, ultrasonography, and cystoscopy help exclude other possible sources, including bladder or upper tract urothelial carcinoma, and can sometimes reveal the source of the hematuria [3]. Retrograde pyelography can detect UIAF only if a favorable pressure gradient exists between the iliac artery and ureter, and this technique is not reliable in the presence of significant ureteral hemorrhage [3], as in our case. Diagnostic ureteroscopy is of little use in this circumstance due to limited visualization [10]. Negative diagnostic findings in any study do not exclude the diagnosis of UIAF, and in cases of uncontrolled bleeding in some patients, surgical intervention is essential [11].

Abdominal CT angiography and diagnostic angiography in our patient failed to demonstrate any definitive evidence of UIAF. Angiography, particularly provocative angiography, has increased sensitivity for detection [5]. Provocative angiography involves the manipulation of a ureteral stent during the investigation to provoke active extravasation at the location of the presumed fistula [5]. In our patient, due to ongoing massive hematuria and shock requiring pressors, provocative testing was not performed as the risks outweighed the benefits. Thus, based on the patient’s history, predisposing factors, retrograde pyelography, and cystoscopic findings, we were able to effectively exclude other sources. The patient was managed with empiric ureteral and external iliac artery stenting, which ultimately resulted in the cessation of hematuria.

A multidisciplinary team comprising urologists, radiologists, and vascular surgeons should be involved in the treatment of a diagnosed or suspected UIAF [11]. The treatment of UIAF has evolved and now involves minimally invasive techniques to allow for healing of the ureter and iliac artery [3]. Endovascular treatment with iliac artery stent grafting is currently the treatment of choice due to less morbidity compared to open techniques, with immediate bleeding control [11]. Stent grafting with embolization remains the most common endovascular treatment [10]. Ureteral stenting can help tamponade the fistula, divert urine away from the fistula, and improve hydronephrosis. Open surgery is generally considered only if endoscopic treatment fails [3].

This case report highlights that radiation cystitis and UIAF can exist concomitantly, and a high index of suspicion is required for the diagnosis of UIAF in patients with predisposing factors and massive incessant hematuria. Although radiographic imaging was not definitive in this case, empiric treatment with ureteral and iliac artery stenting resulted in the immediate cessation of hematuria, confirming the diagnosis of UIAF. It is important to medically optimize these patients during the perioperative period as prevention of refractory shock and other complications postoperatively can help mitigate morbidity and mortality.

## Conclusions

Although rare, UIAF can result in life-threatening hemorrhage; hence, a high index of suspicion is necessary to prevent a delay in diagnosis and reduce morbidity and mortality. A multidisciplinary team should be involved early in the management. Diagnosis is not always apparent radiographically, and incessant gross hematuria in a patient with risk factors and endoscopic suspicion should prompt the empiric treatment of a presumed fistula.
